# Female choice impacts resident male takeover in golden snub-nosed monkeys (*Rhinopithecus roxellana*)

**DOI:** 10.24272/j.issn.2095-8137.2018.035

**Published:** 2018-05-12

**Authors:** Gu Fang, Jing Chen, Ru-Liang Pan, Xiao-Guang Qi, Bao-Guo Li

**Affiliations:** 1 College of Life Sciences, Northwest University, Xi’an Shaanxi 710069, China; 2 Shaanxi Key Laboratory for Animal Conservation, Northwest University, Xi’an Shaanxi 710069, China; 3 School of Anatomy, Physiology and Human Biology, University of Western Australia, Perth Western Australia 6009, Australia

**Keywords:** *Rhinopithecus roxellana*, Female mate choice, Takeover, Resident male tenure, Social network analysis

## Abstract

In primate species with social systems consisting of one-male breeding units (OMUs), resident male takeover represents a major challenge to individual reproductive success and mating strategies. The golden snub-nosed monkey (*Rhinopithecus roxellana*) is characterized by large multilevel societies (MLS) comprised of several OMUs and all-male units (AMUs); however, the factors and mechanisms associated with resident male takeover, which offer important insight into primate reproduction and social strategies, are still poorly understood. Based on 5-year monitoring data from a free-ranging herd of golden monkeys from the Qinling Mountains in China, we categorized three phases of an OMU, that is, a rising phase, developing phase, and declining phase. The rising and declining phases were unstable periods in which male takeover in an OMU might occur. Factors causing takeover, such as leader male rank, fighting ability, reproduction rate, and affiliation (proximity, allogrooming), were analyzed for males and females and for different OMUs. Results indicated that the new resident male’s fighting ability was lower than that of the former resident male in 23 cases. After replacement, the rank order of the new resident male significantly declined. Females involved in a takeover increased their distance from the resident male and decreased mating frequency during the three months prior to takeover. Females with infants under one-year-old had a marked effect on the specific time of takeover occurrence. These results suggested that female choice was the main factor deciding whether a takeover attempt was successful. Furthermore, rather than male conflict, females more often initiated and affected takeover and outcome, implying that the social status and competitive ability of the males played lesser roles during takeover.

## INTRODUCTION

In mammal species with polygamous breeding groups, a new male can enter an established group and forcibly expel the resident adult male (Paul et al., 2000; van Schaik, 2000). This process is called male replacement or takeover (Rudran, 1973; Wheatley, 1982). Resident males monopolize the reproductive behavior of the adult females and maintain their long-term position in the group after male takeover (Inoue & Takenaka, 2008). Correspondingly, females may engage in several alternative behavioral strategies in response to changes in social dynamics during male takeover. For example, female emigration can occur immediately after the collapse of a social group when the previous male leader is ousted (Pusey & Packer, 1987). Alternatively, females may also leave their former unit before conflict or takeover, resulting in possible unit collapse. Therefore, female transfer and mating choice can play important roles for new male mating opportunities (Swedell, 2000).

Resident male migration can influence the social organization, mating strategies, and genetic structure of a species or population (Kuester & Paul, 1999), especially in polygamous primates that form multilevel societies (MLS). Only a small number of primate species, including hamadryas baboons (*Papio hamadryas*), gelada baboons (*Theropithecus gelada*), and snub-nosed monkeys (*Rhinopithecus* spp.), are reported to live in MLS, within which one-male units (OMUs) travel, feed, and rest together to form a cohesive band (Dunbar, 1988; Grüeter et al., 2017). In wild hamadryas baboons, most females show their acceptance of the intruding male following a takeover after a series of behavioral stages (Swedell, 2000). In gelada baboons, however, females residing in the same OMU are closely related, forming matrilines, so males must aggressively compete for reproductive opportunities to maintain their tenure as resident leaders (Dunbar, 1988). In the case of snub-nosed monkeys, the bands are usually followed by one or more all-male units (AMUs) comprised of several adult, sub-adult, and juvenile males (Grüeter & van Schaik, 2009). In the Yunnan snub-nosed monkey (*R. bieti*), a resident male’s rank is very important in regard to successful takeover (Zhu et al., 2016).

Golden snub-nosed monkeys (*R. roxellana*) are an endangered primate species, as evaluated by the IUCN, and are characterized by a polygamous MLS system (Qi et al., 2014, 2017). Several OMUs forage and rest together, forming a breeding band. Bachelor bands formed by ousted males, both former resident males and young males who have reached sexual maturity, are usually found nearby. During the mating season, the bachelor groups are often observed approaching the breeding bands to seek reproductive opportunities (Qi et al., 2014). As a countermeasure, harem males can form alliances to evict solitary males (Qi et al., 2017). Thus, male takeover in *R. roxellana* is influenced by adult males from bachelor groups. It has been reported that conflict occurs during takeover in *R. roxellana* (Wang et al., 2004; Zhao et al., 2011). In addition, harem female preference can affect the takeover process (Qi et al., 2009). However, the specific mechanisms of takeovers, as well as the social, reproductive, and demographic factors that influence the timing and success of male takeovers, remain unclear. Therefore, we carried out a study on the golden snub-nosed monkeys from the Qinling Mountains to clarify the mechanisms of male takeover and hypothesized that male fighting ability had limited impact on takeover events. This study was carried by collecting data on male takeover events, social ranks, and factors that may affect affiliation between males and females before and after takeover, including spatial proximity, grooming, reproductive success, and female birth rate. Our research provides valuable evidence to help understand the mechanism(s) involved in male takeover and reproductive and mating strategies in *R. roxellana*.

## MATERIALS AND METHODS

### Study site

This research was conducted in the Yuhuangmiao region of the Zhouzhi National Nature Reserve (ZNNR), located on the northern slopes of the Qinling Mountains (E108${}^{\circ }$14${}^{\prime }$–108${}^{\circ }$180${}^{\prime }$, N33${}^{\circ }$45${}^{\prime }$–33${}^{\circ }$50${}^{\prime }$, altitude of 1 400–2 890 m a.s.l.), Shaanxi Province, China (Qi et al., 2008). Vegetation in the area includes deciduous broadleaf forest (1 400–2 200 m a.s.l.), mixed coniferous and deciduous broadleaf forest (2 200–2 600 m a.s.l.), and coniferous forest (above 2 600 m a.s.l.) (Li & Zhao, 2007). The region exhibits striking seasonality: the average annual temperature is 10.71 ${}^{\circ }$C, with a maximum of 31.51 ${}^{\circ }$C in July and minimum of −14.31 ${}^{\circ }$C in January, and average annual rainfall is 894 mm (Li et al., 2001).

### Study troop

Two troops reside in the study area (i.e., East and West Range Troops: ERT and WRT) (Li et al., 2000), separated by the Nancha River. The WRT consists of the GNG-herd and DJF-herd. The GNG-herd was the focal study herd and contained a breeding band of 7–13 OMUs and an all-male band. Behavioral observations were made at 0.5 to 50 m, which allowed behavioral patterns as well as individual ages and genders to be recorded under proximity with provisioned food. Hand feeding and physical contact between researchers and animals were avoided, so all behavioral patterns occurred under natural environments and circumstances.

Individual identification was made according to body characteristics, such as pelage coloration, crown hair pattern, scars or evidence of previous injury, and shape of granulomatous flanges on both sides of the upper lip (Qi et al., 2008, 2009).

### Definition of behavior and takeover

We defined resident male replacement as behavior involving aggressive action, including contacting, chasing, and threatening an individual, aggression in response to rejection of the attacked, or counter-aggression (Grüeter, 2004). If the interval between conflict events between two OMUs was longer than 30 s, two conflicts were recorded, otherwise only one conflict was recorded.

### Data collection

The size and composition of the OMUs were recorded in March 2008 after all individuals in the herd were identified. Other data used in this study were collected from March to June, September to the following January, and from August 2012 to December 2016. In total, data included 36 OMUs and 396 monkeys. All individuals in an OMU were scanned during the observation period to clarify whether a takeover had occurred. Agonistic behavior among OMUs was recorded by all occurrence sampling (Altmann, 1974). Allogrooming between males and females was recorded. Copulation, involving mounting and heterosexual genital contact by intromission and pelvic thrusts (Li & Zhao, 2007), was recorded by all occurrence sampling, with the initiator and receiver both identified. We chose one breeding band as the observation target each observation day (1 000–1 400 h) and allogrooming was recorded for 259 d, totaling 906.5 h.

### Data analysis

Individual social rank was scored according to previous research (de Vries, 1998). The birth rate was calculated based on the following formula:

(1) $$BR=\frac{\sum \left(\frac{I_{i}}{F_{i}}\right)}{D}$$

where *I*_i_ is the number of births observed in the reproductive season; *F*_i_ is the number of adult females during the same period; and *D* is the duration observed and recorded by year (Eisenberg et al., 1981).

The reproductive rate in an OMU, as well as male reproductive success, was determined as follows:

(2) $$R=\frac{I}{2F}$$

where *I* is the sum of infants in the OMU involved in a takeover in the first reproduction season; and *F* is the number of adult females without an infant in the replaced OMU.

The frequency of copulation was determined by *C*/*P*/*T*, where *C* is the time observed during the observation period; *T* is the total observation duration of one OMU, recorded by the hour; and *n* is the number of adult females.

We used the independent *t* test to compare differences in the reproduction rates of a harem male in different periods of tenure.

## RESULTS

### OMU male takeover and male-male conflict

From August 2012 to December 2016, we recorded 36 OMUs, of which nine already existed in 2012, 19 underwent restructuring during the study period, and eight remained unchanged. Of the 36 OMUs, 12 females transferred and four OMUs disappeared from the study troop. For the 16 male takeover events, only 13 were thoroughly recorded before and after, with social rank, tenure, and affiliation between females and males documented in detail. Thus, male replacement in these OMUs was considered successful (Table 1).

**Table 1:** Variation of OMU and transfer of former harem males after replacement

Form of replacement	Replacement	Date	Rank status	Injury	Parturition or not${}^{\#}$	Male transfer${}^{+}$
Direct replacement	BZ→TB	2012.8	16→18	No		a
	FZ→LZ	2013.12	10→15	FZ	2/2 0/2	b & a
	PK→VS	2014.2	2→13	VS	2/2 0/2	b
	BB→HT	2013.3	1→8	No	2/3 1/3	b & c
	SX→G3	2013.9	12→12		3/4 1/4	c
	HB→BQ	2015.10				a
	XJ→LD	2015.11		No		b
	PK→KO	2016.9				a
Sequential/indirect replacement	ST→SQ⌃→HK	2012.12	13→14	ST	1/2 1/2	b & c, b & c
	HK→LZ⌃→YH	2013.10	14→15	LZ${}^{*}$		b, b & c
	PK⌃→BG→QS	2016.11				
	JB→WX→ST	2013.10-2014.4	9→15	WX${}^{*}$	1/1 0/1	b
	DXJ→XC⌃→G3	2016.11		DXJ${}^{*}$		b

Capital letters in the Replacement column represent the ID of each individuals. ^*^: Resident males got injury during the first replacement. ^^^: Resident male whose tenure was shorter than 10 days. ^#^: The left row was the number of female who was in reproductive season delivery; the right row was the female who was in reproduction season did not delivery. ^+^: a, emigrated to other troop; b, transferred into AMU; c, rebuild OMU.

Resident male takeover accounted for 60% of OMU changes in our study. Rank order significantly declined in the new OMU compared with the former OMU (independent sample *t* test: *t*=3.073, *df*=7, *P*<0.05), and the reproductive rate of females following takeover was not significantly different than that of females not involved in a takeover (independent sample *t* test: *t*=0.244, *df*=6, *P*>0.05).

**Figure 1:**
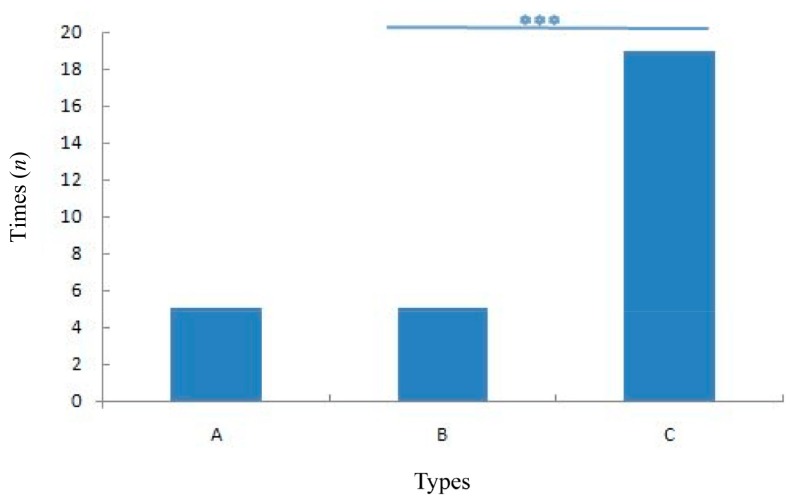
The outcome of replacement fight (five days recording after replacement)

Of the 13 takeovers, only five involved conflict, totaling 23 conflict events. Furthermore, the rate of a new resident male losing the conflict was significantly higher than of it winning (independent sample *t* test: *t*=9.349, *df*=23, *P*<0.001, Figure 1).

Column A: The replacement fight was not observed; B: the times challenged male win during replacement fight; C: the times challenged male lost during replacement fight. ***: *P*<0.001.

### Affiliation of males and females before and after takeover

**Figure 2:**
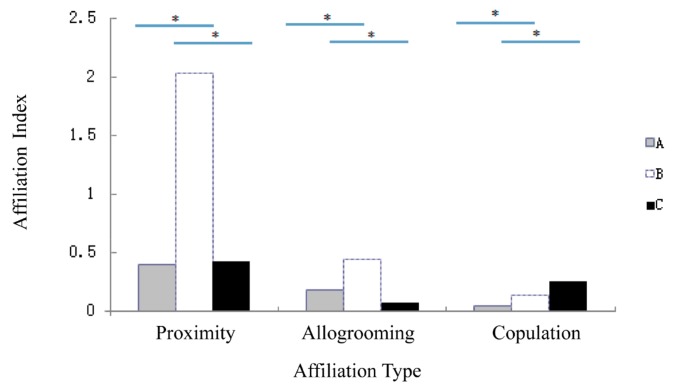
The affiliation between male and female before and after replacement

With regard to the OMUs in which takeovers occurred, affiliation between males and females became weaker in the three months prior to takeover than that observed in OMUs assembled for 3–7 years without takeover (independent sample *t* test: allogrooming: *t*=2.754, *df*=11, *P*<0.05; proximity: *t*=5.149, *df*=11, *P*<0.001; copulation: *t*=8.103, *df*=11, *P*<0.001). Furthermore, for OMUs that experienced a takeover and rebuilt within three months, both allogrooming and proximity were significantly weaker than that observed in OMUs assembled for 3–7 years without takeover (independent sample *t* test: allogrooming: *t*=3.291, *df*=11, *P*<0.001; proximity: *t*=4.052, *df*=11, *P*<0.01); however, mating frequency was significantly higher (independent sample T test: *t*=−11.056, *df*=11, *P*<0.001, Figure 2).

Column A: the OMU which undergo the replacement and rebuild within three months; B: OMU that build 3–7 years; C: the OMU which before the replacement within three months. *: *P*<0.05.

### Resident male tenure affects takeover

According to differences in the number of adult females and infants, as well as the reproductive rates, three distinct OMU development periods were defined: (1) rising phase in the first year; (2) developing phase from years 1–8, during which adult female numbers were stable; and (3) declining phase after 8 years due to a significant reduction in the number of adult females and infants as well as reproductive rates (independent sample *t* test: *t*=−3.254, *df*=38; *P*<0.01).

From 2001 and 2014, the tenure of four of the resident males ranged from 4 to 9 months (average 5.6±2.7, mean±*SD*), whereas the tenure of the other five was 10 years. Seven OMUs stayed in the study troop for more than one year, but less than 8.5 years (average 3.50±2.43, mean±*SD*). Takeover frequency in the first year (rising phase) and tenth year (declining phase) were significantly higher than that in the other years (developing period) (Wilcoxon rank sum test: *Z*=−2.981, *P*<0.01).

### Months of occurrence

There was a significant difference in takeover month between OMUs containing adult females with infants younger than one year and females without young infants (Kruskal-Wallis test: χ${}^{2}$=11.18, *df*=3, *P*<0.05). For OMUs containing females with infants under one-year-old, male replacements occurred from December to the following March, whereas for OMUs comprised of females without young infants, takeovers were random throughout the year.

## DISCUSSION

This study attempted to clarify resident male takeover scenarios in golden snub-nosed monkeys. Our study analyzed factors involved in resident male takeover based on long-term observation and individual identification.

### Limited role of males in takeover

Our results showed that the new resident male often lost the conflict with the former resident male, and social rank order of the newly formed unit was significantly lower than that of the former unit, suggesting that weak conflict ability and lower-ranked males could successfully replace resident males in *R. roxellana*. This result conformed to our previous hypothesis. Specifically, males played a lesser role in the takeover process as former resident males could win the fight but still lose the harem.

In golden snub-nosed monkeys, resident males in breeding bands always face the danger of takeover due to the existence of all-male groups. In these groups, males struggle to gain breeding opportunities, and thus fighting for takeover occurs frequently. However, fierce conflict can lead to substantial injury regardless of winning or losing. In the wild, small injuries can have severe consequences, even resulting in death, which can be a long-term threat to animal populations. Therefore, if takeover outcome is decided by fighting ability alone, it would be harmful for population development during evolution. Thus, *R. roxellana* males do not display fierce conflict during the process of male takeover, as supported by evidence from captive populations. Ren et al. (2007) reported that this not only reduces the probability of injury, but also lowers the cost of male takeover. This behavioral pattern is also seen in other species, including wild barbary macaques (*Macaca sylvanus*) and some group-living mammals (Johnstone, 2000; Nonacs & Hager, 2011).

### Female choice in takeover initiation

Females involved in a takeover increased the distance from and showed an alienated association with the former resident male and decreased mating frequency and mating chances in the three months prior to takeover. Thus, our evidence strongly indicated that male takeover in *R. roxellana* was primarily associated with female behavior changes. Females have the option of direct mate choice by joining or deserting the new male, or by leaving when the resident male is the target of harassment (Sicotte et al., 2017). Male takeovers are largely determined by female transfer and mate choice, which both play critical roles in female social and reproductive strategies (Sterck et al., 1997; van Schaik, 1989). *Rhinopithecus roxellana* females expressed their choice by alienating the former resident male and mating less often, thereby directly initiating and influencing the takeover process.

We also found that mating frequency was significantly higher after the takeover. It may be that females balance the cost of wasting investment in having offspring against the risks of failing to bond with the new male (Mori & Dunbar, 1985). We argue that female mate choice is the primary factor initiating male takeover. Given the limited male sexual coercion and low levels of female aggression, females appeared to select OMUs based on the quality of adult males, which may reduce the ability of the resident male to monopolize access and increase the opportunity of females to mate with different males; however, this requires further research. In our study, mating frequency was significantly higher after male takeover than that before. This may be a way in which females choose mates and increase genetic diversity. Furthermore, mating after male takeover helps to establish good sexual relationships and assists unit members to accept each other and quickly stabilize the OMU. Females likely initiate male takeover to reduce inbreeding and ensure that their offspring are sired by different males over their reproductive lifetime.

### Resident male tenure affects takeover

Our results demonstrated that takeover frequencies in the first year (rising phase) and tenth year (declining phase) were significantly higher than that in the other years (developing period). It has been reported that resident male tenure is the most important factor that negatively affects male reproductive success (Sommer & Rajpurohit, 1989). Females are less likely to be inseminated by males with relatively short or long tenures, which may lead to potential reproductive competition (Qi et al., 2009). Thus, a female more often chooses a male with a suitable tenure (Inoue & Takenaka, 2008). In our case, the reproductive rate after the eighth year was significantly lower than that in the previous years. Relatively short tenure (rising phase) showed unstable affiliation and lower reproductive rates. Adult females may transfer to a more stable environment by takeover to improve female reproduction. Female can also lower potential reproductive competition by transferring.

### Strategies of females with infants

Our results demonstrated significant differences in takeover month between OMUs containing adult females with infants under one year and females without. For OMUs involving females with a young infant, male replacement tended to occur from December to the following March, whereas for OMUs containing females without young infants, male replacement was random throughout the year.

Females carrying infants less than one year old may also influence the seasonal breeding of *R. roxellana*. Related studies show that the golden snub-nosed monkey is a seasonal breeding species, with breeding and mating seasons occurring in March to May and September to December, respectively (Qi et al., 2008). In seasonally breeding populations, the optimal time for takeovers is between the breeding and mating seasons (Cords, 2004). However, the timing of resident male takeovers in *R. roxellana* does not support this prediction, as also reported for *R. bieti* (Cui et al., 2006; Kirkpatrick et al., 1998). However, females with an infant less than one year old transferred to new OMUs during the interval between the mating and breeding seasons. This may be related to the multiple male mating exhibited in this species, which is likely a tactic used to confuse paternity as sneaky mating between females and non-resident males was observed in the present study. Thus, it may be beneficial for females who have a very young infant to initiate takeover during this time.

Although other factors can influence takeover, our results indicated that female choice was the main factor triggering takeover and outcome. A male’s social status and his competitive ability played lesser roles. Female mate choice was another driver that decided whether a male had a chance at successful reproduction.
